# Chronic Mucocutaneous Candidiasis in Autoimmune Polyendocrine Syndrome Type 1

**DOI:** 10.3389/fimmu.2018.02570

**Published:** 2018-11-19

**Authors:** Linda Humbert, Marjorie Cornu, Emmanuelle Proust-Lemoine, Jagadeesh Bayry, Jean-Louis Wemeau, Marie-Christine Vantyghem, Boualem Sendid

**Affiliations:** ^1^Department of Endocrinology and Metabolism, CHU Lille, Lille, France; ^2^Department Parasitology-Mycology, CHU, Lille, France; ^3^Inserm, U995-LIRIC, Fungal Associated Invasive & Inflammatory Diseases, Lille, France; ^4^Department of Endocrinology, Polyclinique Aguilera, Biarritz, France; ^5^Inserm, Center de Recherche des Cordeliers, Sorbonne Université, Université Paris Descartes, Sorbonne Paris Cité, Paris, France; ^6^UMR 1190, Translational Research in Diabetes Inserm, Lille, France; ^7^European Genomic Institute for Diabetes, Univ Lille, Lille, France

**Keywords:** autoimmune polyendocrinopathy candidiasis ectodermal dystrophy (APECED), chronic mucocutaneous candidiasis (CMC), autoimmune regulator (*AIRE*) gene, IL-17, IL-22

## Abstract

Autoimmune polyendocrinopathy candidiasis ectodermal dystrophy (APECED) is an autosomal recessive disease caused by mutations in the autoimmune regulator (AIRE) gene, characterized by the clinical triad of chronic mucocutaneous candidiasis (CMC), hypoparathyroidism, and adrenal insufficiency. CMC can be complicated by systemic candidiasis or oral squamous cell carcinoma (SCC), and may lead to death. The role of chronic *Candida* infection in the etiopathogenesis of oral SCC is unclear. Long-term use of fluconazole has led to the emergence of *Candida albicans* strains with decreased susceptibility to azoles. CMC is associated with an impaired Th17 cell response; however, it remains unclear whether decreased serum IL-17 and IL-22 levels are related to a defect in cytokine production or to neutralizing autoantibodies resulting from mutations in the *AIRE* gene.

## Introduction

Autoimmune polyendocrine syndrome type 1 (APS-1), also known as autoimmune polyendocrinopathy candidiasis ectodermal dystrophy (APECED) syndrome, is characterized by the clinical triad of chronic mucocutaneous candidiasis (CMC), hypoparathyroidism, and adrenal insufficiency. This syndrome was formerly known as Whitaker syndrome (1). Accurate diagnosis of this syndrome requires the presence of at least two of these three major components, or only one if a sibling has already been diagnosed with the disease (2). Other autoimmune disorders have also been described, such as hypergonadotrophic hypogonadism, thyroid disease, type 1 diabetes, coeliac disease, liver disease, alopecia, vitiligo, chronic atrophic gastritis, and hypophysitis. These autoimmune disorders are associated with ectodermal dystrophy, asplenia, and the presence of several autoantibodies, even in the absence of corresponding organ dysfunction (3).

APS-1 is a monogenic, autosomal, recessive disease caused by a mutation in the autoimmune regulator (*AIRE*) gene on chromosome 21 (gene map locus 21q22.3) (4). The *AIRE* gene is composed of 14 exons and codes for a 545 amino acid protein (5, 6). The *AIRE* gene is mainly expressed in thymic medullary epithelial cells, which play an important role in the presentation of self-antigens (7, 8), but is also expressed at low levels in the spleen, lymph nodes, pancreas, adrenal cortex, and peripheral blood mononuclear cells. The *AIRE* gene codes for a nuclear transcriptional regulator protein involved in the ectopic expression of self-antigens in the thymus, leading to the removal of self-reactive thymocytes and generation of peripheral tolerance. The role of peripheral *AIRE* expression, which has been confirmed by mRNA analysis, remains unclear. To date, more than 100 different mutations in this gene, both homogeneous and heterogeneous, have been reported worldwide (9–12).

APECED is a rare syndrome, which has been reported worldwide, but is more prevalent in some historically-isolated homogeneous populations in Finland (1/25000) (4, 13), Sardinia (1/14500) (14), and Iranian Jews (1/9000) (15). APECED is also seen at a lower incidence in Norway, Sweden, Slovenia, Great Britain, Italy, Ireland, and North America (16–21).

Patients with APS-1 suffer from CMC without displaying susceptibility to any other pathogen. CMC is associated with the Finnish mutation c.769C>T (p.Arg257stop) (22). CMC usually affects the oral mucosa, but the nails and skin may also be involved. Esophageal candidiasis results in pain and dysphagia. CMC can be complicated by systemic candidiasis or oral squamous cell carcinomas (SCCs), and may lead to death (23, 24).

## Chronic mucocutaneous candidiasis

CMC is characterized by recurrent or persistent symptomatic mucocutaneous infections caused by *Candida* species, predominantly *Candida albicans*, affecting the nails, skin, oral cavity, and genital mucosa. The diagnosis of CMC is based on clinical symptoms, associated in most cases with the isolation of *Candida* from body sites (25). The first case of syndromic CMC was described by Thorp and Handley (26).

*C. albicans* is a ubiquitous, diploid, dimorphic yeast that resides as a commensal organism on the mucosae and in the gastrointestinal tract of healthy individuals. Mucosal candidiasis results from a change in mucosal homeostasis leading to disequilibrium between the yeast and its host. Opportunistic mucosal infection, deep organ, or systemic infection in immunocompromised patients usually arise from *Candida* colonizing the digestive tract (27). Systemic candidiasis can be diagnosed using a number of non-culture based assays (28) but no biological markers are currently available for the diagnosis of “culture-negative” CMC.

Most cases of CMC are sporadic and are secondary to other medical conditions such as HIV infection with T-cell deficiency, diabetes, immunosuppressive therapies like anti-cytokine blockers, antibiotic or steroid therapy (25, 29, 30). CMC is also more rarely favored by genetic disorders (i.e., familial CMC) that can be inherited. These cases of primary CMC are due mainly to innate immunodeficiency disorders. They have been reviewed by Puel et al. (31) and were classified as primary immunodeficiency disease by the International Union of Immunological Societies Expert Committee for Primary Immunodeficiency in 2015 (32).

Three types of immnuodeficiency can be distinguished depending on the genetic abnormalities associated with CMC:

- Severe underlying immunodeficiency (ID), including severe combined immunodeficiency (SCID), or CD25 deficiency. SCID is a body of diseases characterized by the inability to produce T-cells leading to Th17 cell deficiency (32, 33). Individuals with SCID are susceptible to a whole range of infections caused by bacteria and viruses. Subjects with autosomal recessive CD25 deficiency have a decrease in T-cell and Th17 cell numbers, and a consequent high incidence of viral and bacterial infections (31, 33).
- HIES, CARD9, IL12Rb1 deficiency, GOF-STAT1, and APECED/APS1 are syndromes where CMC has additional specific clinical features. Autosomal dominant hyper IgE syndrome (AD-HIES) is characterized by impaired production of Th17 cells and Th17-derived cytokines caused by an autosomal dominant mutation of *STAT3* (31, 34). AD-HIES is associated with several infectious diseases, including staphylococcal skin abscesses, bacterial pneumonia, and CMC. Recent literature has also described an autosomal recessive hyper-IgE syndrome (AR-HIES), caused by a deficiency in DOCK8, resulting in a variety of symptoms and diseases including atopy, autoimmunity risk, malignancies, recurrent viral/bacterial infections, and CMC. Patients who are deficient in DOCK8 have decreased Th17 cells (35–38). Low concentrations of critical candidacidal peptides, including histatins and β-defensin 2 (BD2), which are activated by IL-17, are found in the saliva of individuals with HIES. Saliva from healthy individuals usually contains high concentrations of β-defensins and histatins, which have direct candidacidal activity. IL-17A, but not IL-22, acts directly on human salivary glands, which might explain why saliva from HIES patients is deficient in histatins and the increased susceptibility of these individuals to CMC. Antimicrobial peptides are secreted by skin epithelial cells only when they are stimulated with Th17 cytokines and classical proinflammatory cytokines (e.g., TNF-α, IL-1β, IFN-γ) (34). Autosomal recessive tyrosine kinase deficiency is another type of combined immunodeficiency that mimics the symptoms of AR-HIES (31–33).
- CMC and invasive *Candida* infection have also been associated with a deficiency in autosomal recessive caspase recruitment domain-containing protein 9 (CARD9) (39–41). CARD9 is an intracellular adaptor involved in Dectin 1 and Dectin 2 signaling, the main pathogen recognition receptors for *C. albicans* glycans. The number of circulating IL-17-producing cells and IL-17 secretion have been reported to be decreased in CARD9-deficient patients (40, 42, 43). However, these findings remain open to debate (33). With regard to *Candida* infections, mutations in Dectin-1 and Dectin-2 have also been studied in murine models. In humans, a mutation in the early-stop codon for Dectin-1 (Y238X) has been reported in a family with recurrent vulvovaginal candidiasis. Experiments *in vitro* demonstrated that monocytes and neutrophils from homozygous patients lacking Dectin-1 expression are defective in cytokine production, including IL-17, when stimulated with *C. albicans*. However, phagocytosis and yeast cell killing remained normal (44, 45). Another report demonstrated that patients receiving hematopoietic stem cell transplants who were heterozygous for Y238X had an increased incidence of gastrointestinal *Candida* colonization (46). Although Dectin-2-deficient mice had higher mortality and a higher kidney fungal burden after infection with *C. albicans* (47), the impact of Dectin-2 mutations on the human host response to *C. albicans* infection remains unclear.
- Other studies have demonstrated that patients with IL-12 or IL-23 signaling defects have an increased risk of developing CMC (31, 33, 48). Patients with CMC, autoimmune manifestations, other mild bacterial or viral infections, intracranial aneurysms, or SCC also had heterozygous missense gain-of-function (GOF) mutations of STAT1. The development of IL-17-producing T-cells is impaired in these patients, as a result of hyperactivity of STAT1 that inhibits STAT3 signaling. This phenomenon results in increased STAT1-dependent cellular responses that repress IL-17-producing T-cell responses, such as IFN-γ, and/or enhanced IL-6, IL-21, and IL-23 STAT1 responses, which normally activate STAT3 and induce IL-17 T-cell production (31).
- Isolated CMC has also been described in subjects with IL-17RA, IL-17RC, ACT1, IL-17F, and RORγt deficiency, where CMC is the only presenting feature of the disease. Some families have also been identified with autosomal-dominant mutations in the gene coding for IL-17F, or autosomal-recessive mutations in the gene coding for IL-17 receptor A or IL-17 receptor C as risk factors for CMC (31–33).
- These familial cases of CMC demonstrate that IL-17 plays a pivotal role in human epithelial immunity to *C. albicans*. Another piece of evidence supporting the central role of Th17 cytokines in CMC and mucosal immunity to yeasts is the observation of cases of CMC in the Phase 2 trial of secukinumab, a human anti-IL17 receptor antibody for the treatment of Crohn's disease (49, 50). This review aims to focus on the characteristics of familial CMC associated with APECED syndrome.

## Chronic mucocutaneous candidiasis and APECED

### Clinical description

CMC is the most common infection occurring in APECED patients (77–100%) (19, 21, 23, 51–53), except in Iranian Jews (17%) (15). CMC is also the most common first clinical manifestation of APECED syndrome (40–93%) (20, 23, 52, 54, 55). Median age at diagnosis is usually <5-years-old (1.0–6.5years) (20, 21, 23, 54, 55). According to the Finnish series, one-sixth of patients had developed CMC by 1.0 year, half by 5 years, 70% by 10 years, 94% by 20 years, and 97% by 30 years of age (56).

The clinical course of CMC varies from periodic to chronic, and its severity varies between individuals. The oral cavity was involved in 100% of patients in the Finnish cohort (23). In the Norwegian cohort, 40% of patients had angular cheilitis (53). In the mild oral form, CMC causes ulceration, redness, and soreness of the corners of the mouth. In more severe cases, the entire mouth is involved making it impossible to consume acidic or spicy foods. In the hyperplastic form, the tongue and buccal mucous membranes are covered by white or gray plaques and hyperkeratosis. In the atrophic form, the mucosa is erythematous and may be speckled with areas of leukoplakic or nodules (56). *Candida* onychomycosis is often associated with mucosal *Candida* lesions in childhood and is very difficult to eliminate (56). CMC affected the nails in 72% of patients in the Irish cohort of Collins et al. (52), and less frequently the skin (10–17%) (21, 23). Esophageal CMC occurred in 5–22% of patients in a European series (23, 52, 53), and in 51% of patients in a recent American study (21). Esophageal candidiasis often occurs without the typical form or symptoms of oral candidiasis, and can be complicated by substernal pain, dysphagia, and stenosis. Esophageal stenosis requires endoscopic dilation (23, 52–54). In the digestive tract, CMC can cause abdominal pain, flatulence, and diarrhea, which may be severe. Symptomatic intestinal candidiasis may also be present in the absence of oral disease (56).

In some patients, CMC may also be complicated by systemic candidiasis, although evidence is lacking that dissemination occurs from the oral cavity. Systemic candidiasis is very rare, even in APECED patients, and is frequently associated with immunosuppressive therapy. In an Italian cohort of 41 patients, one patient died from systemic candidiasis after the onset of immunosuppressive treatment (54), and in a French cohort, one patient died from systemic candidiasis after the onset of immunosuppressive treatment for large granular lymphocytic leukemia (57). In a Finnish cohort, one patient developed an abscess on the pericardium and small intestine (23). One isolated case of chronic *Candida* otitis has been described (53).

### Genotype-phenotype correlation

The prevalence of CMC is reportedly higher in patients with the major Finnish *AIRE* mutation R257X than with other mutations (22). The prevalence is <20% in Iranian Jews affected by the Y85C mutation (15). Kisand et al. studied 160 APECED patients with the most severe mutations, R257X (Finnish) and R139X (Sardinian) and the Norwegian mutation, 967-979Δ13 In contrast to the study of Puel et al. (58), CMC was less prevalent in patients with the homozygous mutation 967-979Δ13 than with the other two mutations (59).

### Squamous cell carcinoma and CMC

CMC has been reported to be involved in carcinogenesis as cancer often develops at the site of fungal lesions. Several cases of oral carcinoma have been described in association with CMC of the oral cavity and esophagus, suggesting that oral candidiasis may be carcinogenic. The most common morphological entity of these cancers is SCC (29, 60, 61). In patients with chronic *Candida* infection, oral CMC is often associated with esophageal cancer (62), and good clinical practice should include regular monitoring, every 2–3 years, by endoscopy. CMC is induced by immunosuppressive therapy rather than SCC itself.

The first report of oral SCC associated with APECED syndrome was published in 1975 in a patient who died of metastatic disease at 27-years of age (63). A case report published in 2010 recorded the presence of three separate oral SCCs in a 40-year-old woman with APECED (61). In the Finnish case series, 6/91 APECED patients >25-years of age (10.5%) developed SCC, four died from the disease, and two developed oral colonization with *Candida* with decreased susceptibility to azole antifungals. One patient developed SCC without symptoms of oral candidiasis (23, 60). In the Norwegian cohort, 3/52 patients (6%) developed SCC at an early age (53). SCC was not reported in a recent American case series (21).

In the general population, mean age at diagnosis for oral and esophageal SCC is >62-years, and the disease is uncommon in young adults (64). The patients diagnosed with oral SCC in the Finnish cohort were between 29- and 44-years of age (23). The high rate of oral SCC in young patients with APECED demonstrates the possible carcinogenic potential of *C. albicans* when associated with the specific immunodeficiencies characteristic of this syndrome. Therefore, appropriate screening and adequate management of the infection and areas of oral dysplasia is necessary to reduce the risk of malignancy. Each erosive, ulcerated lesion should be biopsied, and each dysplastic lesion should be treated surgically (65).

In the general population, there are many risk factors for oral and esophageal SCC. Extrinsic factors include alcohol, tobacco, betel quid, immunosuppression, radiation, oncogenic viruses (human papilloma virus), and *Candida* infection, while intrinsic factors include immune defects, iron or vitamin A deficiency, malnutrition, and defects in tumor suppressor genes (64, 66, 67). Of the six APECED patients who developed SCC in the Finnish cohort, four were smokers and one had received immunosuppressive therapy (23). Therefore, extrinsic factors for SCC should be controlled in APECED patients as well as in CMC.

The role of chronic *Candida* infection in the etiopathogenesis of oral SCC is unclear. Possible mechanisms by which oral *Candida* infection might contribute to cancer development include: (i) metabolism of procarcinogens (such as the conversion of ethanol to acetaldehyde by *Candida*); (ii) production of carcinogens (such as the production of nitrosamine by *Candida* species); and (iii) induction of chronic inflammation, with the production of cytokines that enhance cell proliferation and inhibit apoptosis (24, 64, 65, 68, 69).

### *C. albicans* and decreased susceptibility to antifungal treatment

Oropharyngeal candidiasis (mainly *C. albicans* and *C. glabrata*) is the most common fungal infection in patients with human immunodeficiency virus (HIV), and long-term use of azoles in this population has been reported to cause loss of susceptibility of *C. albicans* to fluconazole (70, 71). Candidiasis is usually caused by the yeast *C. albicans* in APECED patients, unlike in HIV patients (23, 52, 72–75). In a group of Finnish patients with APECED, non-*C. albicans* spp. were reported in only 7/56 patients (12.5%) (72). Because of the high prevalence of CMC in APECED patients and the risk of SCC, lifelong management of candidiasis with antifungal treatment is necessary (76). Topical intermittent treatment is more frequently prescribed than systemic antifungals, which are restricted to periods of severe symptoms and systemic candidiasis. Unlike in HIV patients, APECED patients with CMC treated with fluconazole have a high risk of becoming colonized with *C. albicans* with decreased susceptibility to fluconazole (76). Emergence of resistance does not appear to be a problem during short-term use, as shown in 43 isolates of *C. albicans* from 23 Finnish APECED patients.

Resistance seems to be correlated to the number of years of antifungal drug use, and is mainly due to the use of triazoles. Rautemaa et al. identified *C. albicans* isolates with decreased susceptibility to fluconazole in 11/56 (20%) Finnish patients (72). In the Irish cohort study of 16 patients with APECED, McManus described 11/16 (69%) patients with clinical signs of oral *Candida* infection and oral *Candida* isolates were recovered from 12/16 (75%) patients. Surprisingly, clinical signs suggestive of candidiasis did not always correlate with microbiological evidence of infection, and yeasts were isolated from only 32% of patients. The susceptibility of sequentially recovered isolates to fluconazole and itraconazole was examined; 11/16 (69%) patients had received antifungal treatment with fluconazole or itraconazole. Four of these 11 patients (36%) had isolates that were resistant or had reduced susceptibility to azoles (74).

Multiple mechanisms could lead to azole resistance in *C. albicans*. Some genes of *C. albicans* involved in ergosterol synthesis can be upregulated and in this way confer resistance to azoles, in particular the *ERG11* gene and CDR1, CDR2, and MDR1 encoded efflux pumps. In the study of McManus, acquisition of such mutations was revealed in *C. albicans* strains isolated during the longitudinal follow-up of patients. *C. albicans* strains recovered sequentially from nine Finnish patients were studied. The major molecular mechanisms leading to azole resistance were GOF mutations in *TAC1*, contributing to overexpression of CDR1 and CDR2, point mutations in *ERG11*, and six new *TAC1* mutations were detected (77). To avoid the emergence of azole resistance, McManus et al. proposed only prescribing azole therapy in patients with mycologically-documented *Candida* infection.

### How should CMC be managed in APECED patients?

Husebye et al. suggested that oral infection should be strictly controlled to prevent the development of cancer. These authors highlighted the importance of having good oral hygiene with abstention from smoking and excessive alcohol consumption, and to avoid eating acidic or spicy food, and toothpastes containing powerful whitening agents. Meticulous oral hygiene is recommended in CMC patients, using both toothpaste and chlorhexidine solution, at bedtime and long-term, with the continual use of two topical polyene drugs. In line with this recommendation, patients should hold 1–2 mL of nystatin suspension in their mouth for several minutes and then allow an amphotericin B lozenge to dissolve without chewing. Both drugs should be spread to every part of the mouth with the tongue and finally swallowed. This regimen should be continued for 4–6 weeks or for at least 1 week after the resolution of symptoms. The use of these two polyene antifungal drugs is important in clinical practice and both are well-tolerated due to a lack of absorption from the gut and a low rate of yeast resistance to these drugs.

Azole agents should be restricted to 2–3 courses per year to avoid decreased susceptibility. This treatment can be followed by prophylactic treatment consisting of 1 week of a polyene antifungal every 3 weeks and 1 week of chlorhexidine mouth rinse twice a day, if CMC becomes recurrent. Prophylactic treatment should be administered more frequently if symptoms persist, and even become daily treatment. In the case of failure of antifungal therapy, it is necessary to alert microbiologists to the possibility of a mixed infection and to use chromogenic media to detect mixed species, which would not be picked up with classical Sabouraud glucose agar. It is also recommended that all species isolated undergo antifungal susceptibility testing so that treatment can be adapted accordingly. Angular cheilitis should be treated by application of natamycin, amorolphine hydrochloride cream, or chlorhexidine gel several times a day, continuing for 4–5 days after the corners of the mouth have healed. Miconazole gel can also be used, while bearing in mind the risk of selection of azole-resistant strains. In order to avoid CMC relapses, antifungal treatment should be prescribed simultaneously for oral candidiasis and angular cheilitis, and biopsy of any lesion should be considered if mucositis with ulceration fails to respond to treatment within 2 weeks. *Candida* esophagitis and digestive CMC with diarrhea should also be treated with the same drug regimen for 1 or 2 weeks. If esophagitis persists, esophagoscopy and mycological sampling should be performed. The use of high-dose fluconazole (200–300 mg once a day for 1 week) must be restricted to severe cases and failure of topical therapy. Vaginal *Candida* infection should be treated with a short course of vaginal fluconazole, while fingernail candidiasis is very hard to eliminate and systemic medication is often necessary for 6 weeks. In general terms, prolonged intravenous (IV) antifungal therapy may be required and administered after obtaining expert medical advice (56, 75).

Adjunct immunotherapy with normal pooled immunoglobulin or IV immunoglobulin G (IVIG) in combination with antifungal agents is another treatment option. IVIG is a pooled IgG therapeutic preparation obtained from the plasma of several thousand healthy donors. In addition to its safety profile, IVIG could benefit CMC-associated APECED as a result of multiple mechanisms, including a reduction of inflammation by targeting various arms of the immune system, inhibition of autoantibody production by B-cells, and B-cell anergy (78). Further, antibodies in IVIG to fungal antigens may also help in pathogen neutralization (79, 80). As high-dose IVIG (1–2 g/kg) is known to inhibit Th17 responses *in vitro* (81, 82) and *in vivo*, including experimental allergic bronchopulmonary aspergillosis (78, 83), studies are required regarding appropriate dose of IVIG for CMC-associated APECED.

### Pathophysiology of CMC associated with APECED

**Figure 1:**
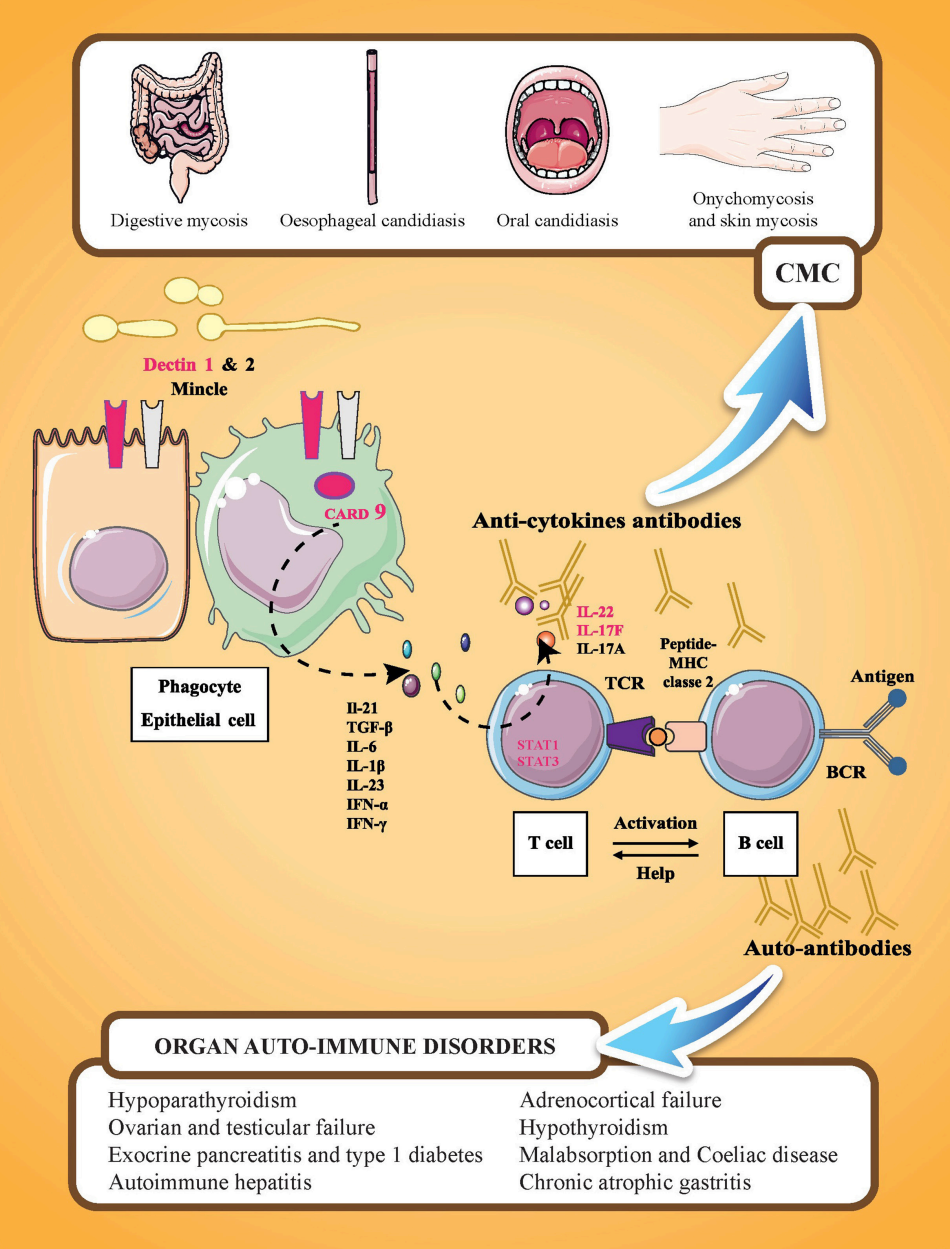
Familial CMC and APECED syndrome. APECED syndrome is characterized by the association of endocrine autoimmune disorders (such as hypoparathyroidism, hypothyroidism, adrenocortical insufficiency, and gonadal failure), non-endocrine autoimmune disorders (such as autoimmune hepatitis, celiac disease, and chronic atrophic gastritis), and chronic mucocutaneous candidiasis (CMC). These manifestations are related to the presence of tissue specific antibodies and cytokine antibodies. IL-17 mediated immunity is represented by cooperation between cells recognizing *C. albicans* (phagocytes and epithelial cells) and IL-17 cytokine-producing cells (T-cells). On *C. albicans* recognition by PRRs (pathogen recognition receptors; including Dectin-1, Dectin-2, or Mincle), the adaptor molecule CARD9 mediates the induction of pro-inflammatory cytokines, such as IL 1β, IL-6, and IL-23. On binding to their receptors expressed on T-lymphocytes, pro-inflammatory cytokines, such as IL-6 or IL-23, activate T-lymphocytes via the transcription factor STAT3 resulting in their differentiation into IL-17-producing T-cells. Genes in which mutations are associated with CMC are indicated in pink: dectin 1, CARD 9, STAT 1, STAT3, IL22, and IL17F. IL-17RA and IL-12RB1 are not represented. Y designates cytokine-neutralizing autoantibodies that develop in AIRE-deficient (APECED and rare thymoma cases) patients.

The most prevalent autoantibodies detected in APECED patients are those neutralizing cytokines, especially type I IFNs and TH17-related cytokines, with a prevalence of 100% for IFN-ω (84, 85) and >90% for IL-22. IFN-ω autoantibodies are also found in thymomas (86), with a high rate of specificity and sensitivity. These autoantibodies are highly disease specific. Antibodies neutralizing IFN-ω are not correlated with CMC, and high titers of anti-IFN-ω antibodies are found in patients without CMC. Antibody titers in patients with APECED are almost always higher at diagnosis and persist for decades, representing a reliable biomarker for APECED syndrome (84, 85). Neutralizing autoantibodies against IL-17A, IL-17F, and IL-22 are also present at diagnosis. Autoantibodies against IL-22 and IL-17F seem to be more prevalent in APECED patients with CMC than in those without CMC, suggesting that type Th17 cytokines are central in human epithelial immunity against *Candida* infection (Figure 1). In contrast to the study of Kisand et al. (59) who reported that autoantibodies neutralizing IL-22 and IL-17F (but not those against IL-17A) were correlated with CMC in a study of 162 APECED patients, a study by Sarkadi et al. (87) reported high levels of autoantibodies against IL-17A in APECED patients with severe CMC. However, recent experimental data have shown that autoantibodies against IL-17A that develop in older *AIRE*-deficient mice do not confer susceptibility to oropharyngeal candidiasis, while monoclonal antibodies that cross-react with murine IL-22 derived from patients increase the mucosal fungal burden (88).

Occasional or weakly binding autoantibodies against IL-6, IL-9, IL-12, IL-21, IL-23, IL-26, IL-29, and RANTES have been reported in APECED patients in addition to autoantibodies against Th17 cytokines; however, their role in the development of CMC has not been demonstrated clearly (59).

The production of type I IFNs by dendritic cells is not impaired in APECED patients (89). *In vitro*, IL-22 and IL-17F production by peripheral blood mononuclear cells of APECED patients, stimulated by *Candida* antigens or polyclonal stimuli, was decreased in patients with CMC (59, 90, 91). The production of IL-17A was not impaired and even appeared to be increased (90, 91). The pathogenesis of CMC is believed to be associated with impaired Th17 cell responses, similar to several other primary immunodeficiencies associated with CMC. Th17 cytokines (IL-17A, IL-17F, and IL-22) influence epithelial cells by inducing the production of chemokines and antimicrobial peptides that exert direct antifungal activity. Additionally, IL-22 promotes epithelial barrier integrity, especially in synergy with TNF-α co-secreted by Th22 cells (92). Moreover, the production of IL-22 is severely impaired by skin-populating T-cells from APECED patients (93).

## Conclusion

In APECED patients, CMC is associated with an impaired Th17 cell response. However, it remains unclear whether decreased serum IL-17 and IL-22 levels are related to a defect in cytokine production or to neutralizing autoantibodies resulting from mutations in the *AIRE* gene. Further investigations to develop new host- or pathogen-derived biomarkers are needed to improve the diagnosis of CMC and for a better understanding of human epithelial immunity against *C. albicans* infection.

## Author contributions

BS and LH conceived the framework of the review. BS, LH, MC, JB, and M-CV wrote the manuscript. EP-L and J-LW revised the manuscript. LH and MC created the Figure. All authors have read and approved the final version of the manuscript.

### Conflict of interest statement

The authors declare that the research was conducted in the absence of any commercial or financial relationships that could be construed as a potential conflict of interest.
